# Conjugates of Small Molecule Drugs with Antibodies and Other Proteins

**DOI:** 10.3390/biomedicines2010001

**Published:** 2014-01-24

**Authors:** Yang Feng, Zhongyu Zhu, Weizao Chen, Ponraj Prabakaran, Kedan Lin, Dimiter S. Dimitrov

**Affiliations:** 1 Protein Interactions Group, Cancer and Inflammation Program, Center for Cancer Research, National Cancer Institute, National Institutes of Health, Frederick, MD 21702, USA; E-Mails: zhongyu.zhu@nih.gov (Z.Z.); chenw3@mail.nih.gov (W.C.); prabakaran.ponraj@nih.gov (P.P.); dimiter.dimitrov@nih.gov (D.S.D.); 2 Basic Science Program, Leidos Biomedical Research, Inc., Frederick National Laboratory for Cancer Research, Frederick, MD 21702, USA; 3 Genentech., San Francisco, CA 94080, USA; E-Mail: lin.kedan@gene.com

**Keywords:** antibody-drug conjugates, ADC, biotherapeutics, drugs, biologics

## Abstract

Conjugates of small molecule drugs with antibodies (ADCs) and with other proteins (protein-drug conjugates, PDC) are used as a new class of targeted therapeutics combining the specificity of monoclonal antibodies (mAbs) and other proteins with potent cytotoxic activity of small molecule drugs for the treatment of cancer and other diseases. A(P)DCs have three major components, antibody (targeting protein), linker and payload, the cytotoxic drug. Recently, advances in identifying targets, selecting highly specific mAbs of preferred isotypes, optimizing linker technology and improving chemical methods for conjugation have led to the approval of two ADCs by Food and Drug Administration (FDA) and more than 30 ADCs in advanced clinical development. However, the complex and heterogeneous nature of A(P)DCs often cause poor solubility, instability, aggregation and eventually unwanted toxicity. This article reviews the main components of A(P)DCs, and discusses the choices for drugs, linkers and conjugation methods currently used. Future work will need to focus on developments and strategies for overcoming such major problems associated with the A(P)DCs.

## 1. Introduction

An ideal anti-tumor drug kills tumor cells efficiently but leaves normal cells intact. With the emergence of antibody-drug conjugates (ADCs) we may be a step closer to this goal. Therapeutic antibodies have exquisite specificity toward antigens that they are designed to recognize. Conjugated with potent cytotoxic drugs, which are otherwise too toxic to use alone, ADCs promise to deliver these cytotoxic drugs directly into tumor cells and release the drug inside cells. Approval of Brentuximab vedotin (ADCETRIS^®^) by US FDA in 2011 has ushered many other similar ADCs into the development for cancer therapy [1,2]. Brentuximab vedotin [3] was approved to treat Hodgkin’s lymphoma (HL) after failure of autologous stem cell transplantation, and systemic anaplastic large cell lymphoma (ALCL) after failure of multi-agent treatment. In February 2013, US FDA approved trastuzumab emtansine (Kadcyla™) for patients with HER2-positive, late-stage (metastatic) breast cancer [4]. It has been reported that currently more than 30 ADCs are in clinical studies [5]. 

This article reviews the main components of ADCs and other protein-drug conjugates (PDCs), and discusses choices for drugs and conjugations by using ADCs currently under late clinical studies as examples. Several recent reviews provide details in efficacy and adverse effects of these ADCs [1,2,6,7,8,9,10]. 

## 2. Antibodies and Targets

Therapeutic antibodies eliminate target cells through two mechanisms in general. One is through triggering signaling from the receptor, which may lead to apoptosis or disrupting signal transduction essential for growth, such as bevacizumab [11]. This mechanism is usually determined by the inherent nature of the antibody’s target. Agonistic antibodies, such as PRO95780, targeting the death receptors are examples of this mechanism [12]. The other mechanism is through the effector function of antibodies, namely antibody-dependent cell-mediated cytotoxicity (ADCC) and complement- dependent cytotoxicity (CDC). Immunoglobulin isotypes IgG1 and IgG3, are capable of mediating these effector functions [13]. However, in many cases, these cell-killing mechanisms are not sufficient to eliminate tumor cells. In addition, some antibodies with high specificity to cancer antigens may lack therapeutic effect by themselves. Conjugating these antibodies to potent cytotoxic drugs is an excellent way to empower these antibodies. 

An appropriate ADC target should have elevated levels on tumor cells and limited expressions in normal cells. For example, Her2 has been shown to overexpress in significant portion of metastatic breast cancers, and with very restricted distribution in normal cells [14,15]. Identifying such cancer differential targets is one of the most important steps in developing ADCs. Higher density of the target molecules could facilitate introduction of more cytotoxic drugs into cells. In addition to selective expression in cancer cells, target molecules should undergo internalization mostly through the early endosome-lysosome pathway [16]. As the targeting moiety of the ADCs, other than characteristics a typical therapeutic antibody should have (e.g., high specificity and high affinity), the antibody preferentially triggers the internalization upon its binding to the cell surface receptor, making ADCs available to intracellular processing [17]. The internalization of antibody/receptor complex may be mediated through the direct engagement of the receptor or clustering of multiple receptors [18,19]. Many receptors, such as G protein coupled receptors (GPCRs), have adapted a mechanism to desensitize to repeated or prolonged exposure by recycling rapidly back to cytoplasmic membrane through early endosomes [20], which makes them less ideal as ADC targets. However, an ADC targeting the GPCR family member endothelin receptor type B (ETBR), Anti-ETBR-MC-vc-PAB-MMAE, has been advanced to clinical development [21], which suggests that unexplored GPCRs with effective internalization could be potential targets for the ADCs [22].

A selected list of ADCs in late stage clinical development and their components along with the details of targets, cytotoxic drugs, therapeutic area, and status is given in Table 1.

**Table 1:** Examples of antibody-drug conjugates in late clinical development (grouped by conjugation methods and drug release mechanisms).

ADC name	Target	Cytotoxic drug	Therapeutic area	Current status
*Linked to cysteines via maleimidocaproyl-VC dipeptide-PAB-MMAE and cleavage by cathepsin B*				
Brentuximab vedotin	CD30	MMAE	HL, ALCL	Approved (2011)
CDX-011 (Glembatumumab vedotin)	GPNMB	MMAE	Breast cancer melanoma	Phase 2
RG-7593 (Pinatuzuumab vedotin) DCDT2980S	CD22	MMAE	DLBL, follicular non-Hodgkin’s lymphoma	Phase 2
PSMA-ADC	PSMA	MMAE	Prostate cancer	Phase 2
*Linked to lysines via SMCC thioether and released by proteolytic degradation of antibody*				
Trastuzumab emtansine	Her2	Maytansinoid DM1	Metastatic breast cancer	Approved (2013)
Milatuzumab-dox	CD74	doxorubicin	Multiple myeloma	Phase 2
*Linked to lysines via acetyl butyrate hydrazone and released by hydrolysis at low pH*				
Gemtuzumab ozogamicin	CD33	N-acetyl- γ calicheamicin	AML	withdrawn
Inotuzumab ozogamincin	CD22	N-acetyl- γ calicheamicin	NHL, ALL	Phase 3
*Linked to lysines via hindered disulfide bond in SPDB and released by reductive cleavage*				
BT062	CD138	maytansinoid	Multiple myeloma	Phase 2
SAR3419	CD19	maytanisinoid	DLBL, ALL	Phase 2
Lorvotuzumab mertansine (IMGN-901)	CD56	maytanisinoid	Small-cell lung cancer	Phase 2 *

Abbreviations: MMAE, monomethylauristatin E; VC, valine citrulline; SMCC, 4-(-maleimidomethyl)cyclohexanecarboxylic acid N-hydroxysuccinimide ester; SPDB, N-succinimidyl 3-(2-pyridyldithiol)butyrate; SPP, N-succinimidyl 4-(2-pyridyldithio)pentanoate; HL, Hodgkin lymphoma; ALCL, anaplastic large-cell lymphoma; AML, acute myeloid leukemia; NHL, non-Hodgkin lymphoma; ALL, acute lymphocytic leukemia; DLBL, diffuse large B-cell lymphoma; GPNMB, glycoprotein nonmetastatic B; PSMA, prostate-specific membrane antigen; * Lorvotuzumab phase 2 studied has been discontinued.

Although the vast majority of reported ADCs employ humanized or fully human antibodies as the guiding molecules, other proteins could perform this function. For instance, soluble forms of the HIV-1 primary receptor CD4 (sCD4) are potentially promising molecules which could target cytotoxic drugs to and kill HIV-1-infected cells expressing the viral envelope glycoproteins (Envs) on cell surface [23]. Two recent studies showed that decreasing the molecular size of sCD4, which typically is composed of either four (D1–4) or two (D1D2) extracellular CD4 domains, to a single domain (D1) and engineering D1 through mutagenesis lead to largely reduced nonspecificity (e.g., interactions with the major histocompatibility complex class II and presumably other targets) of D1 while preserving D1 binding and cross-reactivity with the Envs [24,25]. Therefore, D1 could be superior to D1–4 and D1D2 as a component of the targeting molecules. Interestingly, a class of antibodies targeting the HIV-1 coreceptor-binding site on the Envs (designated as CD4i antibodies) synergize with sCD4 in binding and neutralization because their epitopes are better exposed or formed in the presence of sCD4 [26,27,28]. It has been proposed that sCD4–CD4i antibody fusion proteins could be better than sCD4 or the antibody alone as targeting molecules of not only ADCs or PDCs but also bispecific antibodies and chimeric antigen receptors (CARs) to guide naturally occurring killer cells for HIV-1 eradication [23].

## 3. Cytotoxic Drugs and Mechanisms of Action

It is critical to maintain the antibody pharmocokinetics, antigen binding ability and thermostability of ADCs. There are limited numbers of drug molecules that can be attached to antibody. Too many drug molecules lead to short serum half life of ADCs and increased hydrophobicity [29,30]. The current consensus is three to four molecules of drug per antibody [1,2]. In the early days of protein-drug conjugates, toxins such as ricin were used [31]. The chemotherapy drugs doxorubicin [32,33], methotrexate [34], vinca alkaloids [35], *etc.*, have also been conjugated to antibodies. These drugs were found not potent enough to kill cancer cells, especially in solid tumors where only suboptimal amount of drug are delivered and accumulated. More potent cytotoxic drugs were later used for preparing ADCs. Although these potent cell killing drugs are too toxic to use as free drug, they have been proven to be safe and potent when conjugated with tumor specific antibodies. The drugs used in ADCs currently may be classified into two families: drugs that damage DNA and drugs that disrupt microtubule polymerization [36]. 

Duocarmycin and calicheamicin are two examples of DNA damaging agents. Duocarmycin was first isolated from culture of Streptomyces. It binds to the minor groove of DNA and alkylates the nucleobase adenine, which leads to DNA double strand breaks and apoptosis. It has IC50 of ~10 pM against cancer cells [37]. Synthetic analogs of duocarmycin are available in modified and pro-drug forms for stability requirement before it reaches DNA. MDX-1203, for example, a CD70 targeting ADC conjugated with duocarmycin pro-drug which is under development for treating Non-Hodgkin’s lymphoma and renal cell carcinoma [38]. 

Calicheamicin was first isolated in 1980s from Micromonospora echinospora that live in caliche clay. It belongs to the family of enediyne antibiotics. Calicheamicins bind with DNA in the minor groove, where they then undergo a reaction analogous to the Bergman cyclization, generating a diradical species. The diradical intermediates remove hydrogen atoms from the deoxyribose backbone of DNA which results in strand breaks [39]. Calicheamicin is one of the most potent antitumor agents known. It impacts both quiescent and proliferating cells. N-acetyl-γ calicheamicin is the cytotoxic compound in both Gemtuzumab ozogamicin and Inotuzumab ozogamincin [17]. Gemtuzumab ozogamicin was the first ADC approved by US FDA for acute myeloid leukemia and later withdrawn for safety concerns [40]. Inotuzumab ozogamincin is currently in phase 3 clinical trial. 

Auristatins and maytansinoids are the two most selective microtubule disrupting drugs for ADCs. Both are vinca-domain binders of β-tubulin and prevent polymerization of tubulins into microtubules and disrupt mitotic spindles [36,41]. Therefore, their cytotoxicity is mainly on dividing cells. Monomethyl auristatin E (MMAE), the payload in Brentuximab vedotin, is a synthetic dolastatin 10 derivative. Dolastatin 10 is a lipophilic pentapeptide isolated from the mollusk *Dolabella auricularia* [42]. As an antitumor agent, it is more potent than vinblastine and taxol. Total synthesis of dolastatin 10 was reported in 1989 [43]. Dolastatins may be sensitive to the efflux pump by the multi-drug resistant p-glycoprotein [36]. Monomethyl auristatin F (MMAF) has a charged *C*-terminal phenylalanine residue instead of a carboxyl residue [44]. In comparison with MMAE, MMAF has attenuated membrane translocation capacity, less potent (higher maximal tolerated dose), and much higher aqueous solubility. ADCs comprising MMAE include the FDA approved Brentuximab vedotin [3], and other ADCs that are in late stage clinical development such as Glembatumumab vedotin [45], Pinatuzuumab vedotin [46] and PSMA-ADC [47]. ADCs targeting CD30 [44] and CD22 [48] have the MMAF as a payload component which exhibited different properties and activities as compared to ADCs anti-CD30-MMAE and CD22-DM1 respectively.

Maytainsine is a type of ansamycin antibiotics originally isolated from Ethiopian shrub Maytenus serrate, which inhibits tubulin polymerization and binding at a rhizoxin binding site. Trastuzumab emtansine (T-DM1, Kadcyla^®^) targeting the human epidermal growth factor receptor (HER2) which was approved by FDA has the DM1 (derivative of maytansine) as potent cytotoxic component of the ADC [4].

## 4. Linkers and Conjugation Chemistry

The potent cytotoxic drugs are covalently attached to antibodies through linkers, and the linker stability is critical to the efficacy of ADCs. Stability of ADCs before they reach target cells is the key to minimize off-target killing and maximize tumor exposure to drugs. Upon internalization of ADCs into cells, linkers should be labile enough to ensure rapid release of drugs into cytoplasm where they bind to tubulins or DNA molecules. Design of linker/spacer is intricately related to how ADC will exert its cytotoxicity intracellularly. The choice of linker determines the release mechanism of drugs that is often limited by chemistry and biology of cancer cells. 

Heterobifunctional linkers are commonly used in ADCs [49], because they allow attachment of drugs through disulfide bond and amide bond. A typical example of linker, such as 4-(-maleimidomethyl)cyclohexanecarboxylic acid N-hydroxysuccinimide ester (SMCC) has N-hydroxysuccinimide (NHS) ester, which reacts with primary amines to form amide bond, and maleimide group that reacts with sulfhydryl group to form stable thioether bond. Linkers such as N-succinimidyl 4-(2-pyridyldithio)pentanoate (SPP) and N-succinimidyl 3-(2-pyridyldithiol)butyrate (SPDB) allow amide bond on one end and disulfide bond on the other end, which may be cleaved in reductive environment of some cancer cells. Besides, since maytansinoids and MMAE are synthetic analogs of their natural product counterparts, sulfhydryl or amine groups can be added during synthesis, making them adaptable to various conjugation chemistry methods. Therefore, the main conjugation sites on antibodies are cysteines (both native and mutated cysteines) and lysines. Although studies on conjugations through carbohydrates [50,51] and selenocysteines [52] have been reported, they have yet to reach clinical tests. 

**Figure 1:**
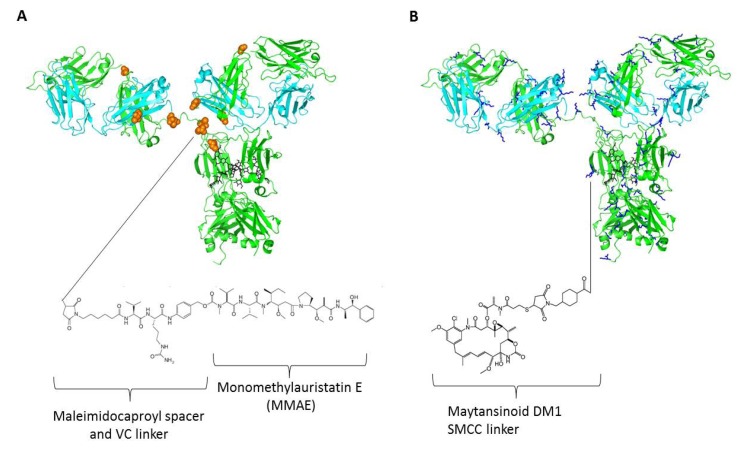
Structural elucidation of antibody drug conjugates (ADCs) using the IgG crystal structure (PDB code: 1HZH) with two-dimensional drawings of drug molecules with linker and spacers (not drawn to scale). (**A**) A model of brentuximab vedotin in which conjugation of cysteines via maleimidocaproyl-VC dipeptide-PAB-MMAE is shown. Residues in orange spheres represent the eight naturally occurring cysteines; (**B**) A model of trastuzumab emtansine in which the drug maytansinoid DM1 is linked to lysines via SMCC thioether. Residues in blue sticks represent the more than eighty naturally occurring lysines.

Cysteine and lysine are two most common naturally occurring amino acids which are used to attach the drug through the linker to the antibody. IgGs have four pairs of interchain disulfide bonds, two between the heavy chains in the hinge region and one on each Fab between CH1 and CL domains (Figure 1A). Intrachain disulfide bonds are to be left intact because they are critical to maintain the basic IgG domain structure essential for antigen recognition, stability and effector functions. Only partial reduction of the interchain disulfide bonds at the hinges gives eight potential conjugation sites. During the conjugation step, molar ratio of free drug and antibody may be adjusted such that approximately four molecules of drugs are attached to each antibody [4,53]. Brentuximab vedotin is conjugated to maleimide-VC-PAB-MMAE on its cysteines [53] (Figure 1A). In the case of Brentuximab vedotin, addition of VC dipeptide provides a proteolytic site for the lysosomal protease cathepsin B to release the drug [54]. Proteolytic activity is abundant in late endosome/lysosomes, such design facilitates rapid release of drug for efficient cell killing. 

The advantage of conjugation through cysteines is the relatively mild condition for reduction and conjugation. The procedure poses minimal risk to the stability of IgGs. The conjugation step is often efficient. There is an established method to monitor the intermediate product and final product ADCs [55]. However, the method gives a mixture of ADCs with 0 to 8 drugs per antibody. To overcome the heterogeneity, site specific conjugation through engineered cysteines, such as THIOMAB [56] has been explored to provide a more homogenous final ADCs with defined drug antibody ratio of 2, better stability and larger therapeutic window. It has been reported that site of conjugation also impacted on ADC stability. Site specific cysteines on heavy, light and Fc regions with various solvent accessibilities have different overall stability and therapeutic index [57]. 

For conjugation through lysines, the primary amine on lysine side chain provides a convenient reactive group for conjugation chemistry. Overall, in an average IgG molecule, there are more than eighty lysine residues and almost a half of them having their side chains exposed to the surface of folded IgG (Figure 1B). More importantly, lysine is not a common residue in the CDR regions which minimizes possible interference of antigen recognition properties while lysine based conjugation of ADCs are used. However, the availability of large number of potential lysine conjugations sites compared to the small number of antibody to antibody ratio (DAR) could lead to a highly heterogeneous mixture. For example, the ADC huN901-DM1 involves about 20 different lysine residues in both light and heavy chains that are found in areas of structural flexibility and with large solvent accessibility [58]. In the recently approved trastuzumab emtansine (T-DM1), the lysine residues on the antibody trastuzumab are connected to a cytotoxic maytansine derivative DM1 through a stable thioether linker N-succinimidyl 4-(Nmaleimidomethyl) cyclohexane-1-carboxylate (SMCC) (Figure 1B). While the N-hydroxysuccinimide ester forms a stable amide bond with the primary amine of any of the available lysine residues of the antibody at pH 7–9, the maleimide moiety undergoes Michael addition reaction with sulfhydryl groups at pH 6.5–7.5 forming stable thioether bonds which are used to attach the drug to the antibody. The conjugation process leads to the formation of a mixture of ADCs with DARs ranging from 0–9, with an average value of 3.5 [59]. The DM1 binds directly to microtubules and inhibits their assembly thereby causing cell death [60]. Although there is no releasing trigger moiety in the T-DM1, following the internalization by the target cancer cells via antigen-mediated endocytosis it was delivered to lysosomes by the vesicular trafficking, and then degraded into the lysine derivatives, lysine-*N^ε^*-SMCC-DM1 [61], which likely bind to the tubulin and cause cytotoxicity by the inhibition of microtubule polymerization.

Lysine conjugation are also used in other ADCs including inotuzumab ozogamincin that is currently in Phase 3 clinical testing for non-Hodgkin lymphoma (NHL) in combination with rituximab [62]. The ADC, inotuzumab ozogamincin has anti-CD22 antibody with 78 lysine residues available for conjugating to cytotoxic drug N-acetyl-γ-calicheamicin via acetyl butyrate hydrazine. The linker drug molecule preferentially attaches to lysines on the heavy chain. The same lysine conjugation as well as drug and linker moieties is used in the other ADC, gemtuzumab ozogamicin (Mylotarg) targeting CD33 which was approved in 2000 by FDA but voluntarily withdrawn in 2010. Both inotuzumab ozogamincin and gemtuzumab ozogamicin use a hydrazone linker connecting through the cytotoxic drug N-acetyl-γ-calicheamicin to the antibody, in which the hydrazone is first cleaved in the acidic intracellular environment followed by the reduction of disulfide bridge in the calicheamicin [63]. Overall, in the case of conjugation through lysine residue, the cytotoxic drug attachment to the antibody typically occurs at a relatively very few ε-amino groups of any of the ~80 lysine residues present in the antibody, because the preferential surface accessibility for the lysine side chains to chemical modification is limited. ADCs with the DAR value of more than certain value, for example more than five, are usually hydrophobic and can lead to solubility problem. Currently, the widely used chemical method for lysine conjugation involves the formation of a stable amide bond using activated ester of the drug molecules, and several methods for the preparation of ADCs through lysine conjugation were described [64]. 

Therefore, both conjugation methods described above are useful and resulting in several ADCs, two of which were approved by the FDA and others are in late clinical development. However, the complexity of ADCs often leads to heterogeneous distribution of drug molecules conjugated to antibodies and unfavorable physicochemical properties including increased aggregation with higher number of DARs, poor aqueous solubility and low stability in circulation. 

## 5. Conclusions

The two recently FDA approved ADCs and growing number of ADCs in the pipeline present promising opportunities in treating various cancers and other diseases. The main optimization parameters in the development of successful ADCs include selection of targets, specificity of mAbs, drug potency, linker stability, and conjugation methods (inter-chain or engineered cysteines, lysines, and Fc glycans). Receptors or cell surface proteins that are overexpressed on tumor cells as compared to normal tissue are good candidate targets. In addition to the usual requirement of therapeutic mAbs, the mAbs used in ADCs must have sufficient aggregation-resistance to withstand the hydrophobicity brought on by adding the drug molecules, and maintain stability and affinity for targets upon conjugation. The selection of antibody isotype, the ability for internalization and tumor penetration are also important. The cytotoxic drugs used in ADCs should be rapidly released in active forms in the intracellular environment. The number of drug molecules in ADCs depend on the method of conjugation, desired potency and linkers [65]. The linkers used in ADCs are a key component, which influences the stability of ADCs and release of drug molecules [66]. Therefore, the choice of linkers used in ADCs profoundly affects the thermal stability, half life, toxicity and the overall efficacy of the ADCs [67]. In order to render better control on the drug/linker conjugation sites on antibodies and the desired DAR, better linker and conjugation methods are needed, as well as rational approaches for improving physicochemical properties and therapeutic index of ADCs. 

## References

[1] Sievers E.L., Senter P.D. (2013). Antibody-drug conjugates in cancer therapy. Annu. Rev. Med..

[2] Lambert J.M. (2013). Drug-conjugated antibodies for the treatment of cancer. Br. J. Clin. Pharmacol..

[3] Francisco J.A., Cerveny C.G., Meyer D.L., Mixan B.J., Klussman K., Chace D.F., Rejniak S.X., Gordon K.A., DeBlanc R., Toki B.E. (2003). cAC10-vcMMAE, an anti-CD30-monomethyl auristatin E conjugate with potent and selective antitumor activity. Blood.

[4] Lewis Phillips G.D., Li G., Dugger D.L., Crocker L.M., Parsons K.L., Mai E., Blattler W.A., Lambert J.M., Chari R.V., Lutz R.J. (2008). Targeting HER2-positive breast cancer with trastuzumab-DM1, an antibody-cytotoxic drug conjugate. Cancer Res..

[5] Mullard A. (2013). Maturing antibody-drug conjugate pipeline hits 30. Nat. Rev. Drug Discov..

[6] Litvak-Greenfeld D., Benhar I. (2012). Risks and untoward toxicities of antibody-based immunoconjugates. Adv. Drug Deliv. Rev..

[7] Mei M., Thomas S., Chen R. (2013). Management of relapsed or refractory hodgkin lymphoma with second-generation antibody-drug conjugates: Focus on brentuximab vedotin. BioDrugs.

[8] Perez H.L., Cardarelli P.M., Deshpande S., Gangwar S., Schroeder G.M., Vite G.D., Borzilleri R.M. (2013). Antibody-drug conjugates: Current status and future directions. Drug Discov. Today.

[9] Bidard F.C., Tredan O. (2013). Trends in cancer-targeted antibody-drug conjugates. Target Oncol..

[10] Ho R.J., Chien J. (2013). Trends in translational medicine and drug targeting and delivery: New insights on an old concept-targeted drug delivery with antibody-drug conjugates for cancers. J. Pharm. Sci..

[11] Presta L.G., Chen H., O’Connor S.J., Chisholm V., Meng Y.G., Krummen L., Winkler M., Ferrara N. (1997). Humanization of an anti-vascular endothelial growth factor monoclonal antibody for the therapy of solid tumors and other disorders. Cancer Res..

[12] Camidge D.R., Herbst R.S., Gordon M.S., Eckhardt S.G., Kurzrock R., Durbin B., Ing J., Tohnya T.M., Sager J., Ashkenazi A. (2010). A phase I safety and pharmacokinetic study of the death receptor 5 agonistic antibody PRO95780 in patients with advanced malignancies. Clin. Cancer Res..

[13] Bindon C.I., Hale G., Bruggemann M., Waldmann H. (1988). Human monoclonal IgG isotypes differ in complement activating function at the level of C4 as well as C1q. J. Exp. Med..

[14] Mandler R., Kobayashi H., Hinson E.R., Brechbiel M.W., Waldmann T.A. (2004). Herceptin-geldanamycin immunoconjugates: Pharmacokinetics, biodistribution, and enhanced antitumor activity. Cancer Res..

[15] Kokai Y., Cohen J.A., Drebin J.A., Greene M.I. (1987). Stage- and tissue-specific expression of the neu oncogene in rat development. Proc. Natl. Acad. Sci. USA.

[16] Ritchie M., Tchistiakova L., Scott N. (2013). Implications of receptor-mediated endocytosis and intracellular trafficking dynamics in the development of antibody drug conjugates. mAbs.

[17] Ricart A.D. (2011). Antibody-drug conjugates of calicheamicin derivative: Gemtuzumab ozogamicin and inotuzumab ozogamicin. Clin. Cancer Res..

[18] Polson A.G., Calemine-Fenaux J., Chan P., Chang W., Christensen E., Clark S., de Sauvage F.J., Eaton D., Elkins K., Elliott J.M. (2009). Antibody-drug conjugates for the treatment of non-Hodgkin’s lymphoma: Target and linker-drug selection. Cancer Res..

[19] Alley S.C., Okeley N.M., Senter P.D. (2010). Antibody-drug conjugates: Targeted drug delivery for cancer. Curr. Opin. Chem. Biol..

[20] Koenig J.A., Edwardson J.M. (1997). Endocytosis and recycling of G protein-coupled receptors. Trends Pharmacol. Sci..

[21] Sassoon I., Blanc V. (2013). Antibody-drug conjugate (ADC) clinical pipeline: A review. Methods Mol. Biol..

[22] Kast J., Boyd R., Ackroyd J., Allen J., Anderson A., Barnes M., Bozhenok L., Dusanjh P., Hudson L., Yu X. (2012). Proteomics highlights which G-protein coupled receptors are candidates for ADC development. Proceedings of the 103rd Annual Meeting of the American Association for Cancer Research, American Association for Cancer Research.

[23] Chen W., Ying T., Dimitrov D.S. (2013). Antibody-based candidate therapeutics against HIV-1: Implications for virus eradication and vaccine design. Expert Opin. Biol. Ther..

[24] Chen W., Feng Y., Gong R., Zhu Z., Wang Y., Zhao Q., Dimitrov D.S. (2011). Engineered single human CD4 domains as potent HIV-1 inhibitors and components of vaccine immunogens. J. Virol..

[25] Chen W., Feng Y., Prabakaran P., Ying T., Wang Y., Sun J., Macedod C.D., Zhu Z., He Y., Polonis V.R. (2013). Highly potent and broad bispecific multivalent HIV-1 inhibitors based on human CD4 and antibody domains. J. Virol..

[26] Chen W., Zhu Z., Feng Y., Dimitrov D.S. (2008). Human domain antibodies to conserved sterically restricted regions on gp120 as exceptionally potent cross-reactive HIV-1 neutralizers. Proc. Natl. Acad. Sci. USA.

[27] Chen W., Xiao X., Wang Y., Zhu Z., Dimitrov D.S. (2010). Bifunctional fusion proteins of the human engineered antibody domain m36 with human soluble CD4 are potent inhibitors of diverse HIV-1 isolates. Antivir. Res..

[28] Dey B., Del Castillo C.S., Berger E.A. (2003). Neutralization of human immunodeficiency virus type 1 by sCD4–17b, a single-chain chimeric protein, based on sequential interaction of gp120 with CD4 and coreceptor. J. Virol..

[29] Hollander I., Kunz A., Hamann P.R. (2008). Selection of reaction additives used in the preparation of monomeric antibody-calicheamicin conjugates. Bioconjug. Chem..

[30] Hamblett K.J., Senter P.D., Chace D.F., Sun M.M., Lenox J., Cerveny C.G., Kissler K.M., Bernhardt S.X., Kopcha A.K., Zabinski R.F. (2004). Effects of drug loading on the antitumor activity of a monoclonal antibody drug conjugate. Clin. Cancer Res..

[31] Roy D.C., Griffin J.D., Belvin M., Blattler W.A., Lambert J.M., Ritz J. (1991). Anti-MY9-blocked-ricin: An immunotoxin for selective targeting of acute myeloid leukemia cells. Blood.

[32] Aboud-Pirak E., Hurwitz E., Bellot F., Schlessinger J., Sela M. (1989). Inhibition of human tumor growth in nude mice by a conjugate of doxorubicin with monoclonal antibodies to epidermal growth factor receptor. Proc. Natl. Acad. Sci. USA.

[33] Sapra P., Stein R., Pickett J., Qu Z., Govindan S.V., Cardillo T.M., Hansen H.J., Horak I.D., Griffiths G.L., Goldenberg D.M. (2005). Anti-CD74 antibody-doxorubicin conjugate, IMMU-110, in a human multiple myeloma xenograft and in monkeys. Clin. Cancer Res..

[34] Kulkarni P.N., Blair A.H., Ghose T., Mammen M. (1985). Conjugation of methotrexate to IgG antibodies and their F(ab)2 fragments and the effect of conjugated methotrexate on tumor growth *in vivo*. Cancer Immunol. Immunother..

[35] Laguzza B.C., Nichols C.L., Briggs S.L., Cullinan G.J., Johnson D.A., Starling J.J., Baker A.L., Bumol T.F., Corvalan J.R. (1989). New antitumor monoclonal antibody-vinca conjugates LY203725 and related compounds: Design, preparation, and representative *in vivo* activity. J. Med. Chem..

[36] Dumontet C., Jordan M.A. (2010). Microtubule-binding agents: A dynamic field of cancer therapeutics. Nat. Rev. Drug Discov..

[37] Wirth T., Schmuck K., Tietze L.F., Sieber S.A. (2012). Duocarmycin analogues target aldehyde dehydrogenase 1 in lung cancer cells. Angew. Chem. Int. Ed. Engl..

[38] Thevanayagam L., Bell A., Chakraborty I., Sufi B., Gangwar S., Zang A., Rangan V., Rao C., Wang Z., Pan C. (2013). Novel detection of DNA-alkylated adducts of antibody-drug conjugates with potentially unique preclinical and biomarker applications. Bioanalysis.

[39] Zein N., Sinha A.M., McGahren W.J., Ellestad G.A. (1988). Calicheamicin gamma 1I: An antitumor antibiotic that cleaves double-stranded DNA site specifically. Science.

[40] Castaigne S. (2013). Why is it so difficult to use gemtuzumab ozogamicin?. Blood.

[41] Pettit G.R., Herz W., Kirby G.W., Moore R.E., Steglich W., Tamm Ch. (1997). The Dolastatins.

[42] Madden T., Tran H.T., Beck D., Huie R., Newman R.A., Pusztai L., Wright J.J., Abbruzzese J.L. (2000). Novel marine-derived anticancer agents: A phase I clinical, pharmacological, and pharmacodynamic study of dolastatin 10 (NSC 376128) in patients with advanced solid tumors. Clin. Cancer Res..

[43] Pettit G.R., Singh S.B., Hogan F., Lloyd-Williams P., Herald D.L., Burkett D.D., Clewlow P.J. (1989). The absolute configuration and synthesis of natural (−)-dolastatin 10. J. Am. Chem. Soc..

[44] Doronina S.O., Mendelsohn B.A., Bovee T.D., Cerveny C.G., Alley S.C., Meyer D.L., Oflazoglu E., Toki B.E., Sanderson R.J., Zabinski R.F. (2006). Enhanced activity of monomethylauristatin F through monoclonal antibody delivery: Effects of linker technology on efficacy and toxicity. Bioconjug. Chem..

[45] Naumovski L., Junutula J.R. (2010). Glembatumumab vedotin, a conjugate of an anti-glycoprotein non-metastatic melanoma protein B mAb and monomethyl auristatin E for the treatment of melanoma and breast cancer. Curr. Opin. Mol. Ther..

[46] Li D., Poon K.A., Yu S.F., Dere R., Go M., Lau J., Zheng B., Elkins K., Danilenko D., Kozak K.R. (2013). DCDT2980S, an anti-CD22-monomethyl auristatin E antibody-drug conjugate, is a potential treatment for non-Hodgkin lymphoma. Mol. Cancer Ther..

[47] Ma D., Hopf C.E., Malewicz A.D., Donovan G.P., Senter P.D., Goeckeler W.F., Maddon P.J., Olson W.C. (2006). Potent antitumor activity of an auristatin-conjugated, fully human monoclonal antibody to prostate-specific membrane antigen. Clin. Cancer Res..

[48] Stephan J.P., Chan P., Lee C., Nelson C., Elliott J.M., Bechtel C., Raab H., Xie D., Akutagawa J., Baudys J. (2008). Anti-CD22-MCC-DM1 and MC-MMAF conjugates: Impact of assay format on pharmacokinetic parameters determination. Bioconjug. Chem..

[49] Hermanson G.T. (2013). Bioconjugate Techniques.

[50] Ramakrishnan B., Boeggeman E., Qasba P.K. (2007). Novel method for *in vitro* O-glycosylation of proteins: Application for bioconjugation. Bioconjug. Chem..

[51] Ramakrishnan B., Boeggeman E., Manzoni M., Zhu Z., Loomis K., Puri A., Dimitrov D.S., Qasba P.K. (2009). Multiple site-specific *in vitro* labeling of single-chain antibody. Bioconjug. Chem..

[52] Hofer T., Skeffington L.R., Chapman C.M., Rader C. (2009). Molecularly defined antibody conjugation through a selenocysteine interface. Biochemistry.

[53] Lyon R.P., Meyer D.L., Setter J.R., Senter P.D. (2012). Conjugation of anticancer drugs through endogenous monoclonal antibody cysteine residues. Methods Enzymol..

[54] Doronina S.O., Bovee T.D., Meyer D.W., Miyamoto J.B., Anderson M.E., Morris-Tilden C.A., Senter P.D. (2008). Novel peptide linkers for highly potent antibody-auristatin conjugate. Bioconjug. Chem..

[55] Valliere-Douglass J.F., McFee W.A., Salas-Solano O. (2012). Native intact mass determination of antibodies conjugated with monomethyl Auristatin E and F at interchain cysteine residues. Anal. Chem..

[56] Junutula J.R., Bhakta S., Raab H., Ervin K.E., Eigenbrot C., Vandlen R., Scheller R.H., Lowman H.B. (2008). Rapid identification of reactive cysteine residues for site-specific labeling of antibody-Fabs. J. Immunol. Methods.

[57] Shen B.Q., Xu K., Liu L., Raab H., Bhakta S., Kenrick M., Parsons-Reponte K.L., Tien J., Yu S.F., Mai E. (2012). Conjugation site modulates the *in vivo* stability and therapeutic activity of antibody-drug conjugates. Nat. Biotechnol..

[58] Wang L., Amphlett G., Blattler W.A., Lambert J.M., Zhang W. (2005). Structural characterization of the maytansinoid-monoclonal antibody immunoconjugate, huN901-DM1, by mass spectrometry. Protein Sci..

[59] Flygare J.A., Pillow T.H., Aristoff P. (2013). Antibody-drug conjugates for the treatment of cancer. Chem. Biol. Drug Des..

[60] Remillard S., Rebhun L.I., Howie G.A., Kupchan S.M. (1975). Antimitotic activity of the potent tumor inhibitor maytansine. Science.

[61] Erickson H.K., Park P.U., Widdison W.C., Kovtun Y.V., Garrett L.M., Hoffman K., Lutz R.J., Goldmacher V.S., Blattler W.A. (2006). Antibody-maytansinoid conjugates are activated in targeted cancer cells by lysosomal degradation and linker-dependent intracellular processing. Cancer Res..

[62] Fayad L., Offner F., Smith M.R., Verhoef G., Johnson P., Kaufman J.L., Rohatiner A., Advani A., Foran J., Hess G. (2013). Safety and clinical activity of a combination therapy comprising two antibody-based targeting agents for the treatment of non-Hodgkin lymphoma: Results of a phase I/II study evaluating the immunoconjugate inotuzumab ozogamicin with rituximab. J. Clin. Oncol..

[63] Damle N.K., Frost P. (2003). Antibody-targeted chemotherapy with immunoconjugates of calicheamicin. Curr. Opin. Pharmacol..

[64] Brun M.P., Gauzy-Lazo L. (2013). Protocols for lysine conjugation. Methods Mol. Biol..

[65] Ducry L., Stump B. (2010). Antibody-drug conjugates: Linking cytotoxic payloads to monoclonal antibodies. Bioconjug. Chem..

[66] Erickson H.K., Widdison W.C., Mayo M.F., Whiteman K., Audette C., Wilhelm S.D., Singh R. (2010). Tumor delivery and *in vivo* processing of disulfide-linked and thioether-linked antibody-maytansinoid conjugates. Bioconjug. Chem..

[67] Acchione M., Kwon H., Jochheim C.M., Atkins W.M. (2012). Impact of linker and conjugation chemistry on antigen binding, Fc receptor binding and thermal stability of model antibody-drug conjugates. mAbs.

